# Dyslexia in the twenty-first century: a commentary on the IDA definition of dyslexia

**DOI:** 10.1007/s11881-024-00311-0

**Published:** 2024-06-15

**Authors:** Julian G. Elliott, Elena L. Grigorenko

**Affiliations:** 1 University of Durham, Durham, UK; 2 University of Houston, Houston, TX 77004 USA

**Keywords:** Assessment and diagnosis, Dyslexia definitions, IDA definition, Reading disability

## Abstract

In offering a commentary upon the IDA definition, we address its main components in turn. While each is technically accurate, we argue that, when taken together, the definition, or more accurately, the use to which it is often put, becomes problematic. We outline different current conceptions of dyslexia and conclude that the operationalisation of the definition for diagnostic purposes often results in scientifically questionable diagnoses and inadvertently leads to significant educational inequity. We propose a simpler definition that describes the primary difficulty, avoids reference to causal explanation, unexpectedness, and secondary outcomes, and redirects practitioner and policymaker focus to the importance of addressing and meeting the needs of all struggling readers.

## Introduction

‘Dyslexia is a specific learning disability that is neurobiological in origin. It is characterized by difficulties with accurate and/or fluent word recognition and by poor spelling and decoding abilities. These difficulties typically result from a deficit in the phonological component of language that is often unexpected in relation to other cognitive abilities and the provision of effective classroom instruction. Secondary consequences may include problems in reading comprehension and reduced reading experience that can impede growth of vocabulary and background knowledge’ (Lyon et al., 2003, p.2).

What is there not to like about this definition? Produced collaboratively by leading researchers, many preeminent in the field, it was approved by the Research Committee of the International Dyslexia Association, adopted for use in research by the US National Institute of Child Health and Human Development of the National Institutes of Health, and is the foundational definition underpinning the subsequent massive proliferation of dyslexia-related legislation in the USA and across the world (Elbeheri & Siang, 2023).

What is there not to like about the definition’s key components? It notes that developmental dyslexia concerns specific problems—accurate and/or fluent word recognition and spelling. It rightly states that there is typically a neurobiological basis to the problem. It highlights phonological deficits as significant factors and notes that these may often not be expected, given the individual’s other cognitive skills or educational experience. It concludes by pointing out that reading problems of this kind can result in reduced reading experience, impaired reading comprehension, vocabulary, and general background knowledge. Surely, no one can take issue with this?

What is there not to like concerning the value of this definition for informing research, promoting practice, and creating policy (Dickman, 2017)? Actually, there is rather a lot not to like. This is not because the definition is scientifically inaccurate or misleading—it is not—but because its interpretation and operationalisation have caused lay, professional and researcher confusion, misunderstanding, and misrepresentation. This has resulted in diagnostic processes and formal labelling that, by catering only to the few, have led to significant educational and social inequity.

## Understandings of dyslexia

The first problem with the definition is that it describes a phenomenon (dyslexia) that is understood, interpreted, and addressed, in a variety of ways (Elliott, 2020). For some, this term operates as a synonym for all types of severe and persistent reading disability; for others, it describes a diagnosable reading (and spelling) difficulty that applies only to a subset of struggling readers. A third group contends that dyslexia describes a different way of thinking; here, reading difficulties are seen as just one of the many ways that the condition can be manifested.

### Dyslexia as a synonym for reading disability

The majority of reading researchers employ the term synonymously with reading disability (Lopes et al., 2020), or, perhaps more precisely, as a ‘word-level reading disability’ (Fletcher et al., 2019). Here, dyslexia is generally understood as being marked by low performance on a normal distribution of reading ability (Seidenberg, 2017). Such difficulties would typically be present from an early age and prove difficult to remedy even with high-quality teaching (Snowling et al., 2020). Where a dyslexia label cut-off should be placed is a largely arbitrary decision that may often reflect administrative policy-related decisions (Snowling, 2019). Where this understanding pertains, we would contend that the IDA definition is largely non-problematic.

If we are emphasising a word-level reading disability, should the definition also include spelling (see, for example, Hall et al., 2022; Pennington et al., 2019)? Given that word reading and spelling skills are closely related processes, at least in alphabetic orthographies, most researchers and practitioners currently include both in their conceptions of dyslexia. Others, however, note that these are not identical skills and a significant proportion of children can experience either a significant reading or a spelling problem (Moll et al., 2014), particularly in the case of more consistent orthographies (Banfi et al., 2022), with co-occurrence between these skills seemingly lower for older students (Kim et al., 2023). Additionally, it has been found that both common and different cognitive predictors apply to early reading and spelling (Sigmund et al., 2024). Kim and Petscher (2023) argue that despite significant overlap, reading and spelling are different and dissociable skills. They note that both are important for literacy development and cite a body of research demonstrating the importance of spelling intervention for the development of word reading.

There are many limitations in our knowledge of reading/spelling relationships. Research studies examining this issue are comparatively few and, to date, have tended not to focus on those participants whose difficulties have persisted despite high-quality intervention. Given these limitations, and the importance of ensuring that a revised definition of dyslexia should have maximum value for guiding educational practice, Elliott and Grigorenko (2024) offer what they consider to be a pragmatic position. Thus, in order to avoid confusion as to the specific nature of an individual child’s literacy difficulties, and to provide the most focused and targeted forms of intervention, reading and spelling deficits should be assessed and identified independently, with the term dyslexia reserved solely to describe problems with accurate and fluent reading. This should not be taken as any diminution of the importance of spelling assessment and instruction. Indeed, we believe that our suggestion will help to reduce any tendency for spelling to be overlooked in educational settings.

### Dyslexia as a specific type of reading problem identified by cognitive testing

A popular understanding is that a clinical distinction should be drawn between the dyslexic and the non-dyslexic poor reader. Those who hold this view would typically argue that the dyslexic individual cannot be identified solely on the basis of the severity of their reading difficulties. To make a differential diagnosis and subsequently ascribe a formal label, assessment needs to be undertaken by a specialist who will typically employ a range of psychometric tests in order to arrive at a diagnosis. Outlining why this position is unsustainable requires a lengthy and detailed explanation that lies outside the scope of this paper but, for such an account, see Elliott and Grigorenko (2024).

### Dyslexia as a different way of thinking

A very different, but increasingly popular, conception sees dyslexia as a ‘…different way of thinking’, that brings with it a range of cognitive strengths and gifts (Davis, 1997; Eide & Eide, 2011; West, 2022), such that it might even be considered to be a desirable difficulty (Gladwell, 2013). Reflecting this perspective, a report produced by a team of management consultants in conjunction with *Made by Dyslexia*, a dyslexia advocacy group, states that:

‘…dyslexic individuals have differing abilities, with strengths in creative, problem solving and communication skills and challenges with spelling, reading and memorising facts. Generally, a dyslexic cognitive profile will be uneven when compared to a neuro-typical cognitive profile. This means that dyslexic individuals really do think differently. What does this mean in work? These varied cognitive profiles give dyslexic individuals natural abilities to form alternative views and solve problems creatively. Heightened cognitive abilities in certain areas, such as visualisation and logical reasoning skills and natural entrepreneurial traits can bring a fresh, often intuitive perspective’ (EY, 2018, p.5).

One can see why such claims would be attractive to many; unfortunately, these have little or no scientific support (Chamberlain et al., 2018; Erbeli et al., 2022; Gilger et al., 2016; Majeed et al., 2021; Martinelli et al., 2018; Seidenberg, 2017). Perhaps, to avoid alienating their membership, misleading messages of this kind often appear to be insufficiently challenged by some dyslexia lobby groups (Johnston & Scanlon, 2021).

## How does the IDA definition help to guide assessment and intervention?

Imagine a dyslexia assessor who is asked to consider the appropriateness of a diagnosis for a child aged 10 years who, it has already been established, has a word-reading age equivalent of 6 years. Having previously ruled out significant sensory or intellectual disability, or the problematics of second language use, on what basis can the IDA definition help the diagnostician to determine whether or not the child is dyslexic? Can the components listed in the definition serve as, or guide the use of, clear criteria? Should the definition even be used for diagnostic purposes? If not, what is its function? Certainly, the definition and its various components are widely used to justify individual clinical decisions although, we would suggest that this is one of the definition’s greatest drawbacks.

The first challenge occurs in the opening sentence. Yes, complex developmental reading difficulties typically have a neurobiological origin [although some have questioned whether this problem can appropriately be termed a neurodevelopmental disorder (Protopapas & Parrila, 2018, 2019)]. However, the wealth of findings from genetic and brain studies of reading disability can lead to an unfortunate misunderstanding that, for any given individual, the aetiology of their reading difficulty can be determined as either predominantly biological (dyslexia) or environmental (a ‘garden variety’ poor reader). Such a belief is wholly inaccurate and the complex interaction of nature and nurture in the development of the reading brain is clearly described in the scientific literature (Church et al., 2021; Elliott & Grigorenko, 2024).

It is to be hoped that simplistic and misleading understandings of this kind will be replaced by increased recognition of the multifactorial nature of reading disability whereby multiple biological and environmental risk and protective factors play a role in the development of the individual’s ‘reading brain’ (Theodoridou et al., 2021; Turesky et al., 2022) and genetically based predispositions change as a consequence of particular environmental contexts (Little & Hart, 2022; Vaughn et al., 2024—this volume).

‘The idea that people are born with dyslexia because they have bad genes and bad brains is an outmoded notion that should be replaced with concepts of risk and malleability that are dependent on instruction and early intervention’ Miciak & Fletcher, 2020, p. 7).

Accepting that reference to dyslexia’s neurobiological origin does not readily permit assessors to make a clinical distinction on the grounds of nature versus nurture, one is left to ponder on how this might otherwise be used to inform diagnosis or intervention.

One might argue that for a small proportion of children, a severe reading difficulty is experienced not because they lack the capacity to learn to read but because they have never been taught to do so (an environmental factor) or, alternatively, because they are unwilling to apply themselves sufficiently (a conative factor). The notion of ‘not being taught’ needs to be interrogated and refined. While there are many illiterate children across the world whose problems are a consequence of a long-term, even lifetime, absence from schooling or some form of alternative educational provision, such individuals are rarely found in dyslexia clinics or require a differential diagnosis of this kind; what they need is sound literacy instruction.

Where regular education is being provided, the key diagnostic issue in relation to schooling may presumably be whether a struggling child has been taught to read using appropriate methods. However, determining whether the child’s reading difficulty is a consequence of poor literacy instruction is often far from easy (Catts et al., 2024—this issue). It is being increasingly accepted that children are likely to make greater progress if closely structured, explicit forms of reading instruction are employed (Hanford, 2018; Seidenberg, 2017), yet such approaches appear to be more essential for some children than others. While whole language approaches, provided in isolation, may not be maximally helpful (Tunmer et al., 2015), most children will become accomplished readers whatever the balance of instructional approaches. It is the child already at risk of reading disability for whom the absence of structured, systematic instruction is particularly disadvantageous.

Any suggestion that a child’s severe reading difficulty may primarily be a consequence of a lack of personal application and effort is untenable. The role of motivation in reading disability is complex (Elliott & Grigorenko, 2024), and for many understandable reasons, some struggling readers appear unwilling to persist in their efforts to learn to read (Vaughn & Fletcher, 2021). Teachers of children with reading difficulties have long recognised that motivational problems are more likely to be a consequence than a cause of reading difficulty, an understanding that has been supported by recent research (Hebbecker et al., 2019; Toste et al., 2020).

The suggestion that it is possible to differentiate between a neurobiological and an instructional or conative aetiology to explain a child’s reading difficulty is not borne out by the scientific literature. Reference to neurobiology in the IDA definition has limited value for educational assessment and decision-making—it offers little explanatory power and serves no meaningful diagnostic function (Sand & Bolger, 2019).

### Phonological problems and other cognitive abilities

The third sentence in the IDA definition states that reading and spelling problems, ‘…typically result from a deficit in the phonological component of language that is often unexpected in relation to other cognitive abilities and the provision of effective classroom instruction’. This is technically correct, but unfortunately, in its rigid application to policy and practice, it can lead to exclusion and inequity. Unfortunately, recognition of the important role of phonological awareness in reading development has resulted in an erroneous belief that observable phonological deficits are necessary for a determination of dyslexia (see, for example, Ottosen et al., 2022, for discussion of the implications of this perspective for diagnosis and resourcing in Denmark).

Studies in which phonological deficits have been linked to reading difficulties have generally been correlational or cross-sectional and thus have largely been unable to resolve issues of causality. Additionally, research findings have been somewhat inconsistent across orthographies (Landerl et al., 2022). Despite these problems, it is generally understood that phonological problems typically play a significant role in the difficulties that struggling readers experience (Perfetti et al., 2019). The likely causal pathway has been subject to debate with some claiming a reciprocal relationship between phonological skills and reading ability (Clayton et al., 2020) with phonological deficits, at least in part, a consequence of reduced or suboptimal reading experience (Huettig et al., 2018).

Despite its primacy in explanatory accounts, a simple causal association has now been largely rejected ‘…because a single phonological deficit is neither necessary nor sufficient to cause the (reading) disorder’ (Pennington et al., 2019, p. 167). It is recognised that ‘…not every person with dyslexia has a phonological deficit’ (Snowling, 2019, p. 55) (see also Dębska et al., 2022; Carroll et al., 2016; Mundy & Hannant, 2020; O’Brien & Yeatman, 2021; Pennington et al., 2012; Valdois et al., 2021), and some children with poor phonological abilities nevertheless develop good reading skills (Bishop, et al., 2009; Catts et al., 2017; Ramus et al., 2013; Ring & Black, 2018).

Rather than employed as a direct marker of reading difficulties, phonological (amongst other) deficits may be better conceptualised as endophenotypes, heritable traits that operate between the genotype and the behavioural phenotype (Moll et al., 2013; Snowling, 2008). These can be present in an individual irrespective of whether, or not, the particular condition (e.g., reading disability) is observed. Their presence increases the risk for an individual of a given disorder but does not guarantee this.

Acceptance of the multifactorial explanation of reading difficulty does not invalidate the emphasis on phonological deficits in the IDA definition. However, the definition is employed to justify inappropriate approaches to assessment and diagnosis. Arguing that a dyslexia diagnosis must be conditional upon the presence of a demonstrable phonological weakness is not only scientifically problematic; it has serious implications for struggling readers who do not present with this problem and who, as a result, could be excluded from special accommodations and resources (Brady, 2019; Pennington et al., 2019; Protopapas & Parrila, 2018).

### Unexpectedness

Potentially, the most problematic reference in the definition concerns the term ‘unexpected’. Originally a North American conception, but more recently gaining in popularity elsewhere, its deployment in the definition reflects the seminal work of Kirk (1963) in the field of specific learning disability. In 2018, the notion of unexpected underachievement was codified in U.S. federal law (U.S. Public Law 115–391). It is widely employed as a key criterion in the diagnosis of learning disability and is also used to differentiate between dyslexic and other struggling readers (Wagner & Lonigan, 2022).

The IDA definition’s reference to unexpectedness has resulted in a degree of ambiguity and confusion. It states that phonological problems are often unexpected in relation to other cognitive abilities and the provision of effective instruction, yet accounts in the dyslexia literature and in professional contexts often suggest that it is the poor reading itself, rather than phonological deficits, that is supposedly unexpected.

The emphasis upon unexpectedness in the assessment of learning disability in general, and reading disability in particular, is rather puzzling, particularly as this criterion can present a major obstacle to the achievement of social and educational equity. As discussed by Elliott and Grigorenko (2024), factors commonly employed to determine whether the reading difficulty is unexpected include family history, IQ, vocabulary, other forms of cognitive ability, strengths and difficulties in other curricular subjects, speech and language difficulties, educational and environmental disadvantage (see discussion of nature v. nurture above), poor schooling, and the presence of internalising and externalising disorders. Such considerations are highly likely to work against the interests of struggling readers who are socially or economically disadvantaged or from minority backgrounds (Chapman & Tunmer, 2019; Knight & Crick, 2021; Odegard et al., 2020; Schatschneider & Hart, 2024).

It has long been the case that those with greater socioeconomic advantage are more likely to seek, and be able to obtain, a dyslexia diagnosis (Kirby & Snowling, 2022; Kirby, 2020a, 2020b). Advantaged families typically have access to cultural capital and the financial resources that enable them to act more fully as advocates for their reading-disabled children (Nevill et al., 2023) and gain access to specialist assessments and resources (Sternberg & Grigorenko, 1999). In the UK, for example, parental challenge can result in school districts (‘local authorities’) being mandated by judicial tribunals to cover the full costs of attendance of diagnosed dyslexic students at expensive independent schools (Elliott et al., under review). Socioeconomic advantage also extends beyond the child’s immediate family; those with reading difficulties who attend schools in affluent neighbourhoods, are more likely to stand out, appear exceptional, and be deemed to have an unexpected problem (Odegard et al., 2020).

Diagnosticians might respond to criticisms about possible bias between unexpectedness and disadvantage by emphasising that their decisions are determined largely on the basis of the individual’s performance on carefully selected tests in carefully structured interviews. When asked to specify precisely the criteria that can differentiate a poor reader with ‘unexpected’ dyslexia from another poor reader who is reading at the same level but not dyslexic, responses usually take one of three forms:

i. The dyslexic individual’s difficulty is not a consequence of severe sensory impairment, intellectual disability, or a discrepancy between their first language and the language of instruction
ii. In the case of the dyslexic individual, discrepancies in performance on various cognitive and academic tests can be identified
iii. While there are no straightforward, easily identifiable criteria, the clinical expertise of the skilled assessor enables them to make a considered, holistic judgement

One might justifiably argue that reading difficulties are unexpected should the individual not present with a severe sensory or intellectual disability, and the language of instruction and assessment is consistent with their home language. The presence of major sensory or intellectual difficulty would already be obvious to all and other forms of specialist assessment and support are required. In the case of a child unfamiliar with the language of instruction, detailed ongoing assessment of their language and reading skills is essential yet, in such a case there would surely be no diagnostic or educational value, in testing for dyslexia.

Vellutino et al.’s (2004) seminal review of the dyslexia literature advised clinicians to:

‘…shift the focus of their clinical activities away from emphasis on psychometric assessment to detect cognitive and biological causes of a child’s reading difficulties for purposes of categorical labelling in favour of assessment that would eventuate in educational and remedial activities tailored to the child’s individual needs’ (p. 31).

Two decades later, there continues to be a significant disconnect between the substantial research literature that has repeatedly confirmed the wisdom of Vellutino et al.’s guidance (Elliott & Grigorenko, 2024; Fletcher & Miciak, 2017; Fletcher et al., 2019; McGill et al., 2018) and much current assessor practice which continues to employ cognitive testing for the purpose of diagnosing learning disability and dyslexia (Al Dahhan et al., 2021; Benson et al., 2019, 2020; Farmer et al., 2021; Kranzler et al., 2020; Lockwood & Farmer, 2020; Lockwood et al., 2022; Maki & Adams, 2019; Sadusky et al., 2022). While outside the scope of this commentary paper, there are many reasons why practitioners might be ignorant of, or choose to disregard, the scientific evidence and continue with empirically and educationally unjustifiable practices (see Elliott & Grigorenko, 2024, for an account).

Recourse to vaguely delineated ‘clinical expertise’ as a substitute for valid, explicit criteria, as the basis for judgement as to whether a given struggling reader’s difficulties are unexpected introduces a significant potential for multiple forms of bias. This may be further influenced by a strong desire to help clients who are clearly struggling with their learning (Suhr & Johnson, 2022). Lilienfeld et al. (2007) describe as an alchemist’s fantasy:

‘…the belief that disparate pieces of data which are invalid on their own are somehow transformed into clinically important information when combined with other data by the expert clinician’ (Harrison & Sparks, 2022, p.271).

Recognising the weaknesses of the responses outlined above, other researchers have applied the term ‘unexpected’ to describe a failure on the part of an individual to make age-related progress in reading, given a standard educational diet (Lachmann & Bergström, 2023) or, alternatively, to a failure to respond sufficiently when provided with additional remedial assistance, for example, within a Response to Intervention (RTI)/Multitier Systems of Support (MTSS) programme (Miciak & Fletcher, 2020; Vaughn et al., 2024—this issue). Here, it is the child’s intractability to reading progress, despite the help subsequently provided, that is deemed to be unexpected. A definition based upon the child’s insufficient instructional response is laudable and offers a powerful way to ensure that the needs of any child who fails to make adequate progress can be recognised and addressed. However, bringing in the notion of unexpectedness in relation to this phenomenon seemingly adds little of value to educational policy or practice.

The final section of the IDA definition lists various secondary consequences which ‘…. may include problems in reading comprehension and reduced reading experience that can impede growth of vocabulary and background knowledge’. Their increased likelihood on the part of struggling readers is indisputable (Fletcher et al., 2019), but it is hard to understand how citing these or other similar consequences can inform individual diagnostic decision-making or educational practice. If a struggling reader were to demonstrate disproportionately high levels of reading comprehension, vocabulary, or general knowledge, would this reduce the readiness of an assessor to diagnose dyslexia in a struggling reader? Indeed, on the contrary, many assessors might argue that such discrepancy would be unexpected and thus serve as evidence to justify a dyslexia diagnosis.

Conceptions of dyslexia as a clinical condition that applies to a subgroup of struggling readers, and which requires extensive psychometric testing by expert clinicians to identify, are outdated and fail to reflect developing understandings about the true nature of reading disability. Rather than offering simplistic unitary explanations, contemporary understandings, as noted above, recognise reading disability/dyslexia as multifactorial with risk factors acting probabilistically, rather than deterministically, along different developmental pathways (Catts & Petscher, 2022; Wagner et al., 2023). Such understandings, incorporating all levels of analysis from genes to environment, no longer permit the simple dyslexic/non-dyslexic binary that has often been associated with the IDA definition.

So, what is the problem with this definition? Simply that, while each element in isolation is accurate, this account singularly fails to identify, or protect the needs of, all struggling readers. The definition can be used to create a false binary whereby a small proportion of more advantaged individuals acquire the dyslexic label and access all the benefits that accrue. While its use of qualifying terms such as ‘typically result’, ‘often unexpected’, and ‘may include’ is consonant with the multifactorial nature and manifestation of reading disability, the inherent uncertainty and subjectivity involved offer a significant advantage to those who have the wherewithal and means to gain access to a diagnostic assessment.

A criticism of Elliott and Grigorenko’s (2014) reference to dyslexia’s conceptual flaws is that such weakness is equally true for many other developmental and psychiatric disorders (see Cutting, 2014; Snowling, 2015; Kirby & Snowling, 2022). Ignoring the fact that this is a poor defence to justify the continuance of a problematic process, it should be noted that dyslexia, when used to differentiate between struggling readers, has a unique weakness. Other conditions, such as those in DSM-5 (American Psychiatric Association, 2013), offer explicit criteria to guide diagnosis which, in turn, enables the formulation of appropriate forms of intervention. Neither of these is true for dyslexia which, as a direct synonym for reading and spelling problems, was only introduced into DSM-5 as a ‘specifier’ following intensive lobbying (see, Elliott & Grigorenko, 2024). A second problem concerns the vast number of people who struggle with reading. Given that as many as 25% of children in advanced industrialised countries may have reading difficulties (Pennington et al., 2019), and such difficulties need to be tackled at as early an age as possible, it is inconceivable that a highly expensive, time-consuming, bottlenecked process of individual diagnosis can be an appropriate means of tackling what is essentially an educational problem with an educational solution.

Drawing upon this (or any other similar) definition to focus on the few, rather than the majority, as is too often the case, is unacceptable. A revised understanding is needed, one that will help to tackle the mass problems of illiteracy that face us across the world. As Odegard et al. (2021) note, the fundamental problem is not dyslexia, it is the fact that large numbers of children in the US (and in other industrialised nations) struggle to acquire even basic reading skills.

## Recommendations

For all the unfortunate misunderstandings and inequities that surround the construct of dyslexia, the term has a popularity and resonance that maintains and sustains its usage. In recognition of its widespread use for referencing poor reading and its often inappropriate diagnostic usage, Elliott and Grigorenko (2024) seek to offer a way forward. Their suggestion would enable the term to be employed in the scientific, practitioner, and lay circles while also offering an understanding that would result in greater consistency and equity in assessment, intervention, and resourcing.

Our recommendation is that the term dyslexia should be defined as:

‘…a severe and persistent difficulty in accurate and fluent word reading’

Dyslexia should be recognised as primarily an educational problem with an educational solution (at least until such time that research demonstrates otherwise), and its deployment as a quasi-medical diagnosis and any reference to its being unexpected, should be discontinued. As such, the term would return to its Greek etymological roots as a descriptor of poor word recognition/decoding; one that moves from a conception that it is an ‘…entity that *causes* poor reading’ to more simply a ‘…*name* for poor reading’ (Protopapas, 2019, p. 8).

Given the probabilistic, multifactorial nature of reading disability/dyslexia, we contend that the use of the term should carry no assumptions as to aetiology (see also, Snowling & Hulme, 2024- this issue). However, the reference in the IDA definition to ‘other cognitive abilities’ has been seen by some as having value in highlighting cognitive factors that appear to play an important role for many poor readers. Catts et al., (2024—this issue), for example, recommend retaining a reference in any future definition to ‘…potential underlying causal factors to assure early identification and intervention to address difficulties beyond reading’ (p. 5). This is likely to result in confusion for assessors and teachers who may seize on particular factors as indicative of the condition. Phonological and oral language deficits, for example, are important contributory factors for many, but not all, struggling readers, and it behoves teachers of young children to monitor progress in these areas. However, as is a common thread throughout this article, references to such factors do not permit differential diagnosis with respect to dyslexic/non-dyslexic poor reader groupings.

While accepting the additional perspective of Catts et al. (2024) that a definition with a causal explanation that could guide the use of meaningful aptitude-treatment interventions would be valuable, we take the view that this is a goal for the future rather than a present reality. Our suggestion, therefore, avoids any direct reference to comorbidities or likely consequences. While researchers and practitioners should be aware and mindful of the need to identify and address these in relation to an individual’s needs, their presence alongside the reading difficulty itself should not be seen to be indicative of an underlying dyslexic condition.

It should no longer be considered appropriate to divide struggling readers into diagnosable dyslexic and non-dyslexic categories or to base resourcing decisions upon the bestowment of this label. Reading ability/disability is a continuous variable, and given the availability of finite resources, formal education systems are likely to continue to need to make decisions about who should receive additional help for reading difficulties. Where required by national or regional education systems for the purposes of resource-based decision-making, formal labelling of difficulties in academic areas should employ a broad classificatory term such as learning disability, learning disorder, or specific learning disability. This should specify the particular areas of difficulty encountered by the individual (accurate and fluent word reading), reading comprehension, spelling, math, etc.). The advantage here is that such terms can be defined and used differentially to meet the individual needs of educational systems across the world.

Unless and until research shows otherwise, in advanced, mature education systems it seems most equitable to base educational resourcing decisions on the severity and persistence of the individual’s problem, perhaps operating within a well-designed and effective RTI/MTSS intervention framework (see Fletcher & Miciak, 2024, for a detailed discussion of such an approach in relation to the determination of specific learning disabilities). Where this approach is undertaken and resourced appropriately, it is unclear how introducing a dyslexia diagnosis (Vaughn et al., 2024—this issue) could add significant value for guiding further forms of intervention.

Elliott and Grigorenko (2024) add a number of further recommendations:

- The assessment of reading difficulties should focus primarily on relevant literacy skills and how these can best be enhanced. As noted above, this does not imply that the assessment of other skills, and processes should not also be undertaken as this may help to provide a broader understanding of the nature of the individual’s learning difficulties
- The expanded understanding of the dyslexia construct, progressing far beyond reading disability, sometimes offering unfounded claims of compensatory abilities or gifts, should be discontinued
- The professional training of educators should include a focus on the prevention of, recognition of, and intervention for, literacy difficulties
- The notion that those hitherto diagnosed as dyslexic require an instructional approach that is different from that appropriate for other struggling readers should be actively dispelled

What is not to like about our definition? Some will argue that it is not helpful for the purposes of differential diagnosis or for determining eligibility for special educational services. We would respectfully respond by agreeing wholeheartedly. In offering a stripped-down definition, we seek a return to the original meaning of dyslexia, one that emphasises a significant difficulty in learning to read, rather than what it has become—a questionable diagnostic label that services the legislative and administrative requirements and constraints of particular educational systems.
